# Ultrasound-guided endoscopic endonasal resection of sellar and suprasellar craniopharyngiomas

**DOI:** 10.3389/fsurg.2023.1073736

**Published:** 2023-02-17

**Authors:** Guilherme Finger, Kyle C. Wu, Sanyia S. Godil, Ricardo L. Carrau, Douglas Hardesty, Daniel M. Prevedello

**Affiliations:** ^1^ Department of Neurosurgery, The Ohio State University College of Medicine, Columbus, OH, United States; ^2^ Department of Otolaryngology and Skull Base Surgery, The Ohio State University Wexner Medical Center, OH, United States

**Keywords:** craniopharyngioma, skull base, transnasal endoscopic surgery, ultrasonography, endoscopic ultrasonography (endoscopic US)

## Abstract

**Introduction:**

Optimal planning and minimally invasive surgical approach are essential to complete craniopharyngiomas (CP) resection with limited postoperative morbidity. Given the nature of craniopharyngioma recurrence, complete resection of the neoplasm is crucial. Since CP arise from the pituitary stalk and may grow anteriorly or laterally, some cases require an extended endonasal craniotomy. The extension of the craniotomy is crucial to expose the whole tumor and to make its dissection from the surrounding structures feasible. In order to guide the extension of the approach, the intraoperative use of ultrasound is helpful for the surgeons. The objective of this paper is to describe and to demonstrate the applicability of the utilization of intraoperative ultrasound (US) guidance for planning and confirmation of craniopharyngioma resection in EES.

**Method:**

The authors selected one operative video of a sellar-suprassellar craniopharyngioma gross-totally resected by EES. The authors demonstrate the extended sellar craniotomy, the anatomic landmarks that guide bone drilling and dural opening, the aspect of the intraoperative real time US, tumor resection and dissection from the surrounding structures.

**Results:**

The solid component of the tumor was mostly isoechogenic in texture compared to the anterior pituitary gland, with several wide spread hyperechogenic images corresponding to calcifications and hypoechogenic vesicles corresponding to cysts inside the CF (“salt-and-pepper” pattern).

**Discussion:**

The intraoperative endonasal US is a new surgical tool that allows for real-time active imaging for skull base procedures, such as sellar region tumors. Besides tumor evaluation, the intraoperative US helps the neurosurgeon to determine the size of craniotomy, to anticipate the relation between the tumor and vascular structures and to guide the best strategy for gross-total resection of the tumor.

**Conclusion:**

The EES allows a straight access to the craniopharyngiomas located in the sellar region or that grow anteriorly or superiorly. This approach allows the surgeon to dissect the tumor with minimal manipulation of the surrounding structures, when compared to craniotomy approaches. In order to accomplish that, the use of intraoperative endonasal ultrasound helps the neurosurgeon to perform the most suitable strategy, optimizing the rate of success.

## Introduction

Craniopharyngioma (CP) is a benign tumor (classified as grade 1 by the World Health Organization) that originates from the coatings of the Rathke's pouch in the sellar–parasellar region (1, 2). Despite the non-malignant histology patterns, the tumor's proximity, and sometimes invasiveness, to adjacent neuro and vascular structures may turn the surgery challenging (1–3).

Endoscopic approaches to the sellar region and expanded endonasal craniotomies (exposing the anterior fossa) represent a less invasive and morbid alternative for CP resection; allowing high rates of gross total resection when applied appropriately (4). In order to achieve proper exposure of the tumor it is mandatory to identify its topography and extent of growth. Hoffman et al. (5) and Samii et al. (6) published two classifications that help to better understand those aspects, which help the neurosurgeon to perform the appropriated craniotomy in each particular case. These classifications are based on preoperative Magnetic Resonance images (MRI), but no paper has described the image characteristics and the applicability of intraoperative real time images.

In the first section of this paper the authors performed a short review about craniopharyngioma's image characteristics and surgical treatment. In the second section, the authors will describe the applicability of intraoperative ultrasound (US) for the resection of craniopharyngiomas by the endoscopic endonasal approach, demonstrating the image patterns of the tumor, its relation to the pituitary stalk and its role helping to decide the durotomy extension. Finally, the authors also demonstrate a surgical video showing the applicability of intraoperative US in the endoscopic endonasal resection of craniopharyngiomas.

## Methods

The surgery was performed at The Ohio State University Wexner Medical Center, Columbus, OH, United States. In the video, the authors demonstrate the endonasal nasal septal flap preparation, the extended sellar craniotomy (with resection of the sphenoid planum and the floor of the anterior fossa), the anatomic landmarks that guide bone drilling and dural opening, the aspect of the intraoperative real time ultrasound (demonstrating the craniopharyngioma, the stalk and the anterior pituitary gland), tumor resection and dissection from the surrounding structures, and the vasculo-nervous structures that must be preserved in order to diminish morbidity and mortality.

The intraoperative endonasal US was performed using the BK Medical Bk 5,000 Ultrasound System with the N20P6 Minimally Invasive 6 × 7 mm Transducer.

The images from the surgery and the ultrasound were gathered and edited using a video editing software.

## Results

**Figure 1:**
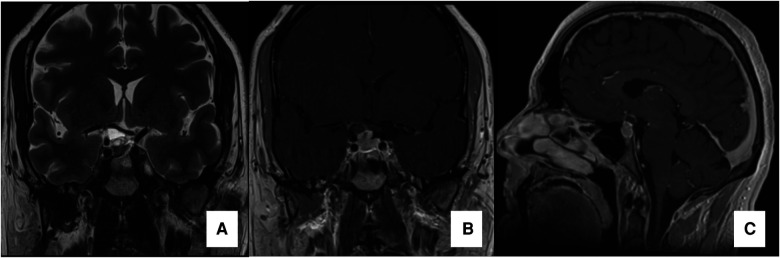
Preoperative MRI preoperative MRI. Coronal T2WI image in (**A**), Coronal and Sagittal T1WI images with gadolinium (**B and C**, respectively) demonstrating a solid-cistic sellar lesion with suprasellar extension compressing the optic chiasm (especially its right portion) and promoting a superior displacement of the first segment of the right anterior cerebral artery.

In the case illustrated in the video, a 40 years-old male presenting a persistent headache whose intensity had progressively increased during the previous 12 months was admitted in the hospital. The neurological exam was unremarkable. A MRI was performed demonstrating a solid-cystic tumor in the sellar region extending to the supra-sellar region. The solid portion of the tumor presented an isointense aspect on T1WI and T2WI with a diffuse but heterogeneous gadolinium enhancement in T1WI (Figure 1). The cystic portion of the tumor presented a hypointense image in T1WI, hyperintense image in T2WI and the capsule of the tumor enhanced after gadolinium infusion.

**Figure 2:**
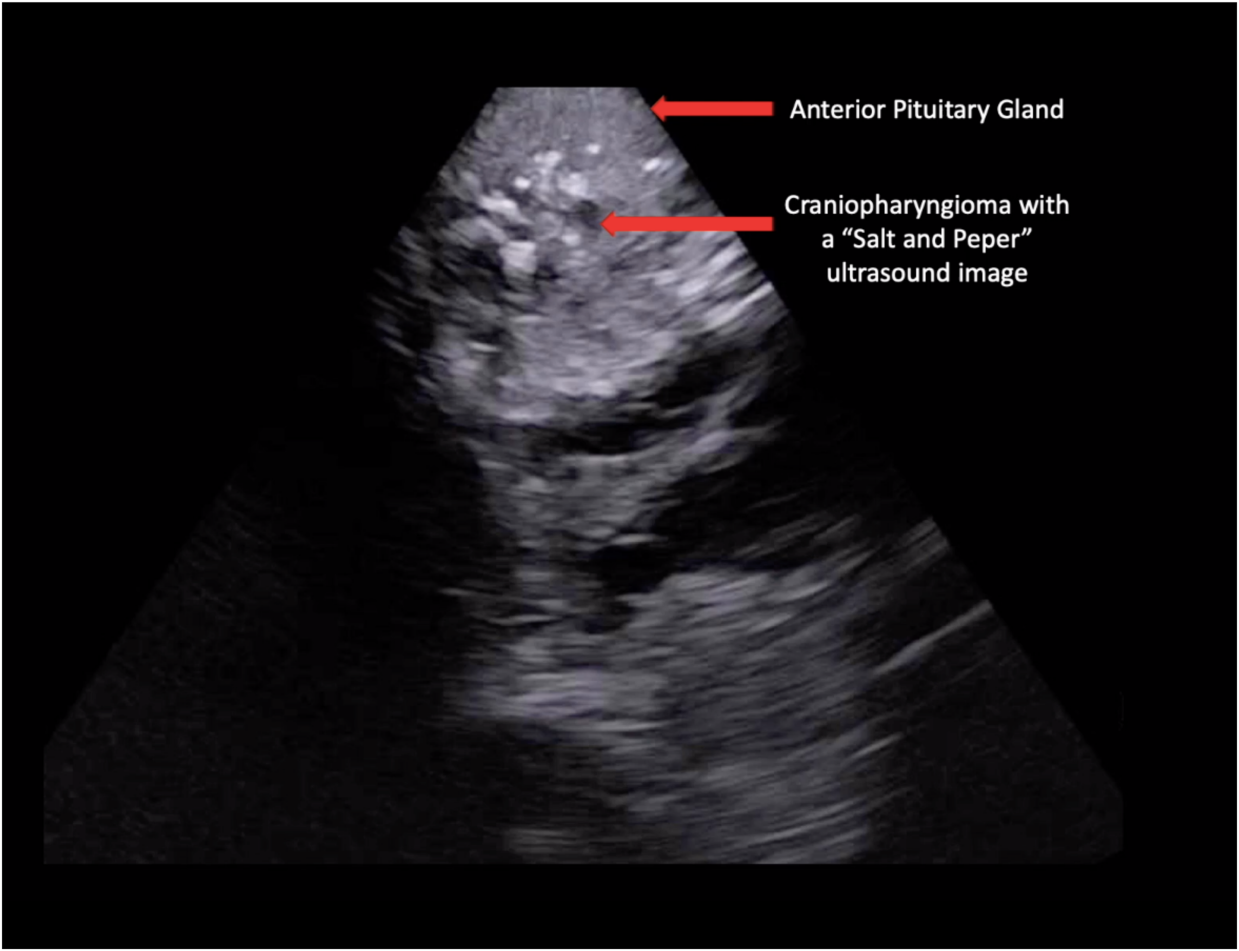
Endonasal sonographic pattern of a craniopharyngioma; intraoperative US picture demonstrating the sonographic characteristics of the craniopharyngioma and its relationship to the anterior pituitary gland.

**Figure 3:**
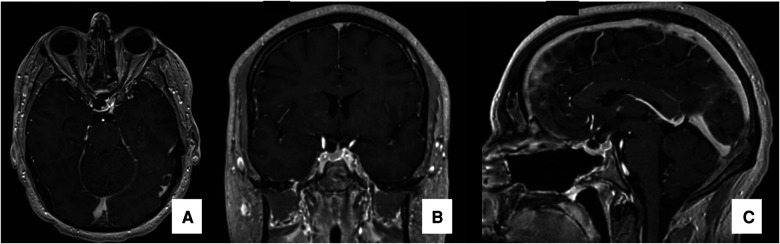
Postoperative MRI postoperative MRI in T1WI images with gadolinium demonstrating gross-total resection of the tumor: axial (**A**), coronal (**B**) and sagittal (**C**) views.

The intraoperative sonographic visualization of the CF demonstrates a heterogeneous tumor with solid-cistic pattern. The solid component of the tumor was mostly isoechogenic texture compared to the anterior pituitary gland, with several wide spread hyperechogenic images corresponding to calcifications and hypoechogenic vesicles corresponding to cysts inside the CF (“salt-and-pepper” pattern) (Figure 2). The pituitary stalk was also visualized and the presence of small calcifications adhered to the stalk were identified. In the video, it is also possible to notice the need for an extended approach (transsphenoidal-transtuberculum) in order to expose the tumor allowing a gross total resection (Figure 3).

During the period of May 1st, 2022 to October 31st, 2022, the endoscopic endonasal ultrasound was utilized in 4 cases of craniopharyngiomas submitted to endoscopic endonasal approach. The endoscopic endonasal US was utilized before dural opening in order to guide the size of the craniotomy, allowing for a complete exposure of the tumor. The relation between the tumor to the pituitary stalk and the surrounding vasculature was also crucial in terms of strategy to resect the tumor. A gross total removal of the tumor was accomplished in all cases, with anatomical and functional preservation of the pituitary stalk and the pituitary gland. The third ventricle and the hypothalamus were not invaded by tumor in these cases. One patient evolved with transitient hypernatremia, recovering from it in the second postoperative day. No vascular or hormonal imbalance were detected.

## Discussion

The CP is usually composed by a solid and cystic component. On imaging, it has typical features that help its diagnosis. Some of these features are best visualized on CT scans while others are best analyzed on MRI.

Computed tomography (CP) is the best method to evaluate the presence of calcifications withing the tumor. Calcium deposits occur in the cystic capsule occur in up to 80% of CP (7, 8). Moreover, the determination of the extent of calcification and the detection of osseous changes in the skull base are also best visualized on CT (4).

The magnetic resonance imaging (MRI) remains the modality of choice for CP evaluation, since it best provides detailed information about the tumor itself, its relation to surrounding structures and gives information regarding possible complications of the tumor. The relationship of the tumor to its surrounding neurovascular structures includes analysis of the pituitary gland, pituitary stalk, optic nerves, optic chiasm, hypothalamus and anterior cerebral arteries. Complications of large CP include hydrocephalus, invasion of the third ventricle and infiltration of the hypothalamus; which are all well visualized on MRI (4). Regarding the tumor evaluation, the MRI clearly demonstrates the cystic and solid components of the tumor. Since the cystic fluid is high in protein and lipid content, it is often hyperintense on both T1WI and T2WI MRI sequences. The capsule of the cyst and the solid component both present enhancement after gadolinium infusion, however the solid component often presents a reticular enhancement pattern on T1WI images.

Based on the MRI, several CP classifications to evaluate the relation of the tumor to different surrounding structures have been described. With respect to the sella turcica, optic chiasm and the floor of the third ventricle, Hoffman et al. classify the CPs into prechiasmatic, retrochiasmatic, subchiasmatic, and intraventricular craniopharyngiomas (5). Sammi et al. (6), classified the CP based on its origin and vertical projection into 5 grades (grade I = intrasellar or infradiaphragmatic, grade II = occupying the cistern with or without an intrasellar component, grade III = lower half of the third ventricle, grade IV = upper half of the third ventricle and grade V = reaching the septum pellucidum or lateral ventricles). Finally, for pediatric patients, a radiological classification based on the extent of hypothalamic involvement was developed by Puget et al. (grade 0 = none, grade 1 = affected but still visible hypothalamus, grade 2 = hypothalamic structures distorted) (9). However, in the adult population, no similar classification exists.

The rare descriptions of US images for CP have been reported only in fetal cases, in which a tumor located in the sellar/anterior fossa region was diagnosed as an incidental finding during gestational regular exams (10, 11). The obstetric US describes the CP as a rounded shaped centrally located, intracranial tumor surrounded by areas of septated fluid (10). The solid component of the tumor is irregularly echogenic, containing hyperechoic areas similar in echo texture to calcifications but without shadowing. The power Doppler sonography may show perfusion within the mass and displacement of the normal vessels around the tumor surface (11).

Despite its innate surgical difficulty due to CP`s location, advances in microsurgical and skull base techniques have made resection possible in many patients (4). The two main determinants for the outcome of CP patients (in terms to avoid recurrence) are the result of the initial surgical excision and the experience of the neurosurgeon (12). The surgery goal is gross total resection of the tumor, however sometimes it is not feasible due to tumor extent and invasiveness of adjacent structures (13). Hypothalamus infiltration precludes a total resection (14), however if the pituitary stalk is infiltrated, gross total resection is still the best option (15). If the stalk is not infiltrated by the tumor, it should be preserved since its maintenance decreases endocrine dysfunction after surgery. When safe gross total resection is not safe (hypothalamic infiltration, for example), a surgical subtotal resection followed by adjuvant radiotherapy is an alternative with good disease control (4, 16).

Conventional microscopic skull base procedures can traditionally be performed *via* transfacial, transcranial or combined open cranial craniotomies (12). However, based on the purpose of the procedure, cystic aspiration or stereotactic biopsy may be performed.

Due to the development, evolution and increasing use of endoscopic techniques in the past decades, the Endoscopic endonasal surgery (EES) seems to be replacing the classic transcranial approaches (2, 12). The EES allows a better visualization of the tumor and its surrounding structures, consequently a higher percentage of gross total resection have been achieved, associated with reduced morbidity and mortality rates (17–19). Even though the nasal corridor is a safe and direct access to the tumor topography, it is important to consider that not all tumors and locations are amenable to this approach. In general, EES is inappropriate when the tumor extends more than 1 cm beyond the lateral limits of the exposure (4).

The relation of the craniopharyngioma to the sellar diaphragm is essential for the decision to choose a transsphenoidal approach. Purelly infradiaphragmatic craniopharyngiomas or intrasellar tumor that stretch the diaphragm sellae upwards are suitable for a transsphenoidal transsellar surgery (12, 20). On the other hand, infradiaphragmatic CPs with suprasellar extension or solely suprasellar CPs require an extended EES with removal of the tuberculum sellae and the posterior planum sphenoidale, described as the transsphenoidal–transtuberculum sellae approach (21).

The intraoperative endonasal US (BK Medical Bk 5,000 Ultrasound System with the N20P6 Minimally Invasive 6 × 7 mm Transducer) is a new surgical tool that allows for real-time active imaging for skull base procedures, such as sellar region tumors. Besides tumor evaluation, the intraoperative US helps the neurosurgeon to evaluate if the tumor can be properly exposed by a traditional sellar craniotomy or if an extended endonasal skull base craniotomy is required. The relation to vascular structures can be best evaluated using the color doppler feature. Finally, the intraoperative US is an instrument that can be easily integrated to the skull base armamentarium, since it does not prolong the surgery time, can be performed multiple times during the same procedure and can be performed in the same operative room. The current main disadvantages of this method are: the lack of image guidelines to help the surgeons to identify the structures that are being visualized and the limitation of this method to few institutions. The authors believe that these limitations are secondary to the fact that this is a novel surgical instrument whose prototype was approved recently and the equipment was distributed to few institutions in order to be improved. The publication of cases as the one presented by the authors in form of research papers (or printed and video atlas), will contribute to the build the knowledge regarding the US views of the normal anatomy as well as the pathologies involving the sellar region.

## Conclusion

The EES allows a straight access to the craniopharyngiomas located in the sellar region or that grow anteriorly or superiorly. This approach allows the surgeon to dissect the tumor with minimal manipulation of the surrounding structures, when compared to craniotomy approaches. In order to accomplish that, the use of intraoperative endonasal ultrasound helps the neurosurgeon to perform the most suitable strategy, optimizing the rate of success.

## Data Availability

The original contributions presented in the study are included in the article/**Supplementary Material**, further inquiries can be directed to the corresponding author.
